# (*E*)-1-(2,4-Dinitro­phen­yl)-2-[1-(thio­phen-2-yl)ethyl­idene]hydrazine

**DOI:** 10.1107/S1600536811011135

**Published:** 2011-04-07

**Authors:** Hoong-Kun Fun, Patcharaporn Jansrisewangwong, Suchada Chantrapromma

**Affiliations:** a X-ray Crystallography Unit, School of Physics, Universiti Sains Malaysia, 11800 USM, Penang, Malaysia; b Crystal Materials Research Unit, Department of Chemistry, Faculty of Science, Prince of Songkla University, Hat-Yai, Songkhla 90112, Thailand

## Abstract

The mol­ecule of the title compound, C_12_H_10_N_4_O_4_S, is slightly twisted, with a dihedral angle of 8.23 (9)° between the benzene and thio­phene rings. One nitro group is co-planar [O—N—C—C torsion angles = −0.5 (3) and −1.9 (3)°] whereas the other is slightly twisted with respect to the benzene ring [O—N—C—C torsion angles = −5.1 (3) and −5.7 (3)°]. In the crystal, the mol­ecules are linked by weak C—H⋯O inter­actions into screw chains along the *b* axis. The mol­ecular conformation is consolidated by an intra­molecular N—H⋯O hydrogen bond.

## Related literature

For bond-length data, see: Allen *et al.* (1987 ▶). For related structures, see: Chantrapromma *et al.* (2010 ▶); Fun *et al.* (2010 ▶); Jansrisewangwong *et al.* (2010 ▶); Shan *et al.* (2008 ▶). For background to and the biological activity of hydrazones, see: Baughman *et al.* (2004 ▶); Bedia *et al.* (2006 ▶); El-Tabl *et al.* (2008) ▶; Ramamohan *et al.* (1995 ▶); Rollas & Küçükgüzel (2007 ▶). For the stability of the temperature controller used in the data collection, see Cosier & Glazer (1986 ▶).
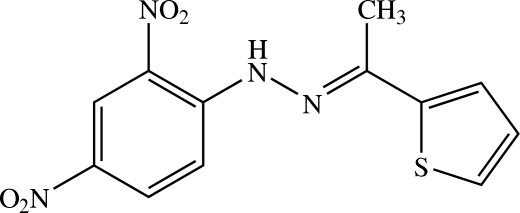


## Experimental

### 

#### Crystal data

C_12_H_10_N_4_O_4_S
*M* *_r_* = 306.30
Monoclinic, 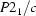

*a* = 9.4868 (5) Å
*b* = 15.3912 (8) Å
*c* = 8.9756 (4) Å
β = 91.672 (2)°
*V* = 1310.00 (11) Å^3^
*Z* = 4
Mo *K*α radiation
μ = 0.27 mm^−1^
*T* = 100 K
0.60 × 0.19 × 0.16 mm

#### Data collection

Bruker APEXII CCD area detector diffractometer
Absorption correction: multi-scan (*SADABS*; Bruker, 2009 ▶) *T*_min_ = 0.854, *T*_max_ = 0.959
10366 measured reflections
2414 independent reflections
2176 reflections with *I* > 2σ(*I*)
*R*_int_ = 0.027

#### Refinement

*R*[*F* ^2^ > 2σ(*F* ^2^)] = 0.041
*wR*(*F* ^2^) = 0.109
*S* = 1.08
2414 reflections
195 parameters
H atoms treated by a mixture of independent and constrained refinement
Δρ_max_ = 0.38 e Å^−3^
Δρ_min_ = −0.44 e Å^−3^

### 

Data collection: *APEX2* (Bruker, 2009 ▶); cell refinement: *SAINT* (Bruker, 2009 ▶); data reduction: *SAINT*; program(s) used to solve structure: *SHELXTL* (Sheldrick, 2008 ▶); program(s) used to refine structure: *SHELXTL*; molecular graphics: *SHELXTL*; software used to prepare material for publication: *SHELXTL* and *PLATON* (Spek, 2009 ▶).

## Supplementary Material

Crystal structure: contains datablocks global, I. DOI: 10.1107/S1600536811011135/rz2573sup1.cif

Structure factors: contains datablocks I. DOI: 10.1107/S1600536811011135/rz2573Isup2.hkl

Additional supplementary materials:  crystallographic information; 3D view; checkCIF report

## Figures and Tables

**Table 1:** Hydrogen-bond geometry (Å, °)

*D*—H⋯*A*	*D*—H	H⋯*A*	*D*⋯*A*	*D*—H⋯*A*
N2—H1*N*2⋯O1	0.79 (3)	2.00 (3)	2.618 (3)	134 (2)
C6—H6*A*⋯O2^i^	0.93	2.45	3.099 (3)	127
C9—H9*A*⋯O4^ii^	0.93	2.47	3.147 (2)	129
C11—H11*A*⋯O3^i^	0.93	2.57	3.240 (3)	129

Symmetry codes: (i) 
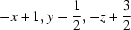
; (ii) 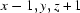.

**Fig. 1.**
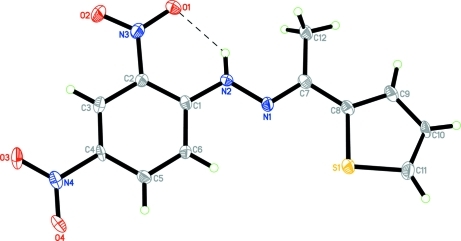
The molecular structure of the title compound, showing 50% probability displacement ellipsoids. The intramolecular hydrogen bond is drawn as dash line.

**Fig. 2.**
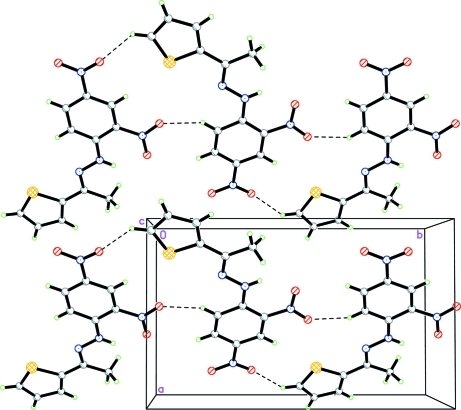
The crystal packing of the title compound viewed along the c axis. Hydrogen bonds are drawn as dashed lines.

## supplementary crystallographic information

### Comment

Hydrazones are an important class of compounds which are used in numerous biological and pharmacological applications as insecticidal, antitumor, antioxidant, antifungal, antibacterial, antiviral and antituberculosis compounds (Bedia *et al.*, 2006; El-Tabl *et al.*, 2008; Ramamohan *et al.*, 1995; Rollas & Küçükgüzel, 2007). Several of them also exhibit good nonlinear optical properties (Baughman *et al.*, 2004). In our previous studies we reported the syntheses and crystal structures of some hydrazone derivatives (Chantrapromma *et al.*, 2010; Fun *et al.*, 2010; Jansrisewangwong *et al.*, 2010). The title compound (I) was synthesized as part of our on going research on biological activities of hydrazones.

The molecule of (I), (Fig. 1), is slightly twisted with the dihedral angle between the benzene and thiophene rings of 8.23 (9)°. The middle ethylidinehydrazine unit (N1/N2/C7/C12) is planar with the *r.m.s*. 0.0033 (2) Å and the torsion angle N2–N1–C7–C12 = -1.1 (3)°. This N—N=C—C bridge makes dihedral angles of 6.62 (11) and 2.14 (12)° with the benzene and thiophene rings, respectively. The nitro group at atom C2 is co-planar [torsion angles O1–N3–C2–C1 = -0.5 (3)° and O2–N3–C2–C3 = -1.9 (3)°] whereby that at atom C4 is slightly twisted with respect to the benzene ring [torsion angles O3–N4–C4–C3 = -5.1 (3)° and O4–N4–C4–C5 = -5.7 (3)°]. The bond distances are of normal values (Allen *et al.*, 1987) and comparable with those found in related structures (Jansrisewangwong *et al.*, 2010; Shan *et al.*, 2008).

In the crystal structure (Fig. 2), the molecules are linked by C—H···O weak interactions (Table 1) into screw chains along the *b* axis. The molecular conformation is consolidated by an intramolecular N—H···O hydrogen bonding interaction (Table 1). C···N^iii, iv^[3.219 (3)–3.232 (3) Å], C···O^iii, v,vi^[3.099 (3)–3.187 (2) Å] and N···O^vii^[2.971 (2) Å] short contacts are also observed [symmetry codes: (iii) *x*, 1/2 - *y*, -1/2 + *z*; (iv) 1 + *x*, 1/2 - *y*, -1/2 + *z*; (v) 1 - *x*, 1/2 + *y*, 3/2 - *z*; (vi) 1 + *x*, *y*, -1 + *z* and (vii) 1 - *x*, 1 - *y*, 2 - *z*].

### Experimental

The title compound was synthesized by dissolving 2,4-dinitrophenylhydrazine (0.40 g, 2 mmol) in ethanol (10 ml) and H_2_SO_4_ (conc.) (98%, 0.5 ml) was slowly added with stirring. Then 2-acetylthiophene (0.20 ml, 2 mmol) was added to the solution with continuous stirring. The solution was refluxed for 30 min yielding an orange-red solid, which was filtered off and washed with methanol. Orange block-shaped single crystals of the title compound suitable for *X*-ray structure determination were recrystalized from ethanol by slow evaporation of the solvent at room temperature over several days. Mp. 516–518 K.

### Refinement

The H atom attached to N2 was located in a difference Fourier map and refined isotropically. The remaining H atoms were positioned geometrically and allowed to ride on their parent atoms, with d(C—H) = 0.93 Å for aromatic and 0.96 Å for CH_3_ atoms. The *U*_iso_ values were constrained to be 1.5*U*_eq_ of the carrier atom for methyl H atoms and 1.2*U*_eq_ for the remaining H atoms. A rotating group model was used for the methyl groups. The highest residual electron density peak is located at 0.97 Å from S1 and the deepest hole is located at 0.67 Å from S1.

### Crystal data

**Table:**

C_12_H_10_N_4_O_4_S	*F*(000) = 632
*M**_r_* = 306.30	*D*_x_ = 1.553 Mg m^−^^3^
Monoclinic, *P*2_1_/*c*	Melting point = 516–518 K
Hall symbol: -P 2ybc	Mo *K*α radiation, λ = 0.71073 Å
*a* = 9.4868 (5) Å	Cell parameters from 2414 reflections
*b* = 15.3912 (8) Å	θ = 2.2–25.5°
*c* = 8.9756 (4) Å	µ = 0.27 mm^−^^1^
β = 91.672 (2)°	*T* = 100 K
*V* = 1310.00 (11) Å^3^	Block, orange
*Z* = 4	0.60 × 0.19 × 0.16 mm

### Data collection

**Table:**

Bruker APEXII CCD area detector diffractometer	2414 independent reflections
Radiation source: sealed tube	2176 reflections with *I* > 2σ(*I*)
graphite	*R*_int_ = 0.027
φ and ω scans	θ_max_ = 25.5°, θ_min_ = 2.2°
Absorption correction: multi-scan (*SADABS*; Bruker, 2009)	*h* = −10→11
*T*_min_ = 0.854, *T*_max_ = 0.959	*k* = −18→14
10366 measured reflections	*l* = −10→10

### Refinement

**Table:**

Refinement on *F*^2^	Primary atom site location: structure-invariant direct methods
Least-squares matrix: full	Secondary atom site location: difference Fourier map
*R*[*F*^2^ > 2σ(*F*^2^)] = 0.041	Hydrogen site location: inferred from neighbouring sites
*wR*(*F*^2^) = 0.109	H atoms treated by a mixture of independent and constrained refinement
*S* = 1.08	*w* = 1/[σ^2^(*F*_o_^2^) + (0.0504*P*)^2^ + 1.3252*P*] where *P* = (*F*_o_^2^ + 2*F*_c_^2^)/3
2414 reflections	(Δ/σ)_max_ < 0.001
195 parameters	Δρ_max_ = 0.38 e Å^−^^3^
0 restraints	Δρ_min_ = −0.44 e Å^−^^3^

### Special details

**Table:**

Experimental. The crystal was placed in the cold stream of an Oxford Cryosystems Cobra open-flow nitrogen cryostat (Cosier & Glazer, 1986) operating at 120.0 (1) K.
Geometry. All e.s.d.'s (except the e.s.d. in the dihedral angle between two l.s. planes) are estimated using the full covariance matrix. The cell e.s.d.'s are taken into account individually in the estimation of e.s.d.'s in distances, angles and torsion angles; correlations between e.s.d.'s in cell parameters are only used when they are defined by crystal symmetry. An approximate (isotropic) treatment of cell e.s.d.'s is used for estimating e.s.d.'s involving l.s. planes.
Refinement. Refinement of *F*^2^ against ALL reflections. The weighted *R*-factor *wR* and goodness of fit *S* are based on *F*^2^, conventional *R*-factors *R* are based on *F*, with *F* set to zero for negative *F*^2^. The threshold expression of *F*^2^ > σ(*F*^2^) is used only for calculating *R*-factors(gt) *etc*. and is not relevant to the choice of reflections for refinement. *R*-factors based on *F*^2^ are statistically about twice as large as those based on *F*, and *R*- factors based on ALL data will be even larger.

### Fractional atomic coordinates and isotropic or equivalent isotropic displacement parameters (Å^2^)

**Table:**

	*x*	*y*	*z*	*U*_iso_*/*U*_eq_	
S1	0.18528 (6)	0.07603 (4)	1.08312 (6)	0.02114 (18)	
O1	0.38055 (16)	0.48642 (10)	0.89817 (17)	0.0234 (4)	
O2	0.5358 (2)	0.53354 (10)	0.7464 (2)	0.0367 (5)	
O3	0.82477 (16)	0.34691 (11)	0.46978 (17)	0.0263 (4)	
O4	0.82742 (17)	0.20890 (11)	0.51711 (18)	0.0295 (4)	
N1	0.29668 (17)	0.24567 (11)	1.00495 (18)	0.0165 (4)	
N2	0.35684 (18)	0.31993 (13)	0.9518 (2)	0.0169 (4)	
H1N2	0.328 (2)	0.3673 (18)	0.967 (3)	0.018 (6)*	
N3	0.47704 (19)	0.47362 (12)	0.8097 (2)	0.0219 (4)	
N4	0.78273 (18)	0.28269 (13)	0.53639 (19)	0.0212 (4)	
C1	0.4601 (2)	0.31302 (13)	0.8510 (2)	0.0150 (4)	
C2	0.5214 (2)	0.38567 (13)	0.7812 (2)	0.0165 (4)	
C3	0.6272 (2)	0.37572 (14)	0.6779 (2)	0.0185 (5)	
H3A	0.6662	0.4239	0.6322	0.022*	
C4	0.6724 (2)	0.29369 (14)	0.6451 (2)	0.0170 (4)	
C5	0.6148 (2)	0.22010 (14)	0.7110 (2)	0.0169 (4)	
H5A	0.6469	0.1649	0.6866	0.020*	
C6	0.5112 (2)	0.23004 (13)	0.8115 (2)	0.0158 (4)	
H6A	0.4731	0.1810	0.8553	0.019*	
C7	0.2011 (2)	0.25572 (14)	1.1041 (2)	0.0166 (4)	
C8	0.1380 (2)	0.17563 (14)	1.1560 (2)	0.0173 (4)	
C9	0.0380 (2)	0.16528 (15)	1.2632 (2)	0.0202 (5)	
H9A	−0.0017	0.2113	1.3144	0.024*	
C10	0.0020 (2)	0.07644 (14)	1.2874 (2)	0.0188 (5)	
H10A	−0.0628	0.0581	1.3565	0.023*	
C11	0.0736 (2)	0.02170 (16)	1.1973 (2)	0.0237 (5)	
H11A	0.0630	−0.0384	1.1977	0.028*	
C12	0.1551 (2)	0.34159 (14)	1.1648 (2)	0.0211 (5)	
H12A	0.2364	0.3740	1.1986	0.032*	
H12B	0.0941	0.3321	1.2468	0.032*	
H12C	0.1054	0.3736	1.0880	0.032*	

### Atomic displacement parameters (Å^2^)

**Table:**

	*U*^11^	*U*^22^	*U*^33^	*U*^12^	*U*^13^	*U*^23^
S1	0.0239 (3)	0.0203 (3)	0.0198 (3)	−0.0021 (2)	0.0103 (2)	−0.0022 (2)
O1	0.0255 (8)	0.0191 (8)	0.0262 (8)	0.0024 (6)	0.0095 (7)	−0.0037 (6)
O2	0.0487 (11)	0.0151 (9)	0.0478 (11)	−0.0032 (8)	0.0247 (9)	0.0050 (8)
O3	0.0254 (8)	0.0333 (10)	0.0206 (8)	−0.0092 (7)	0.0095 (6)	0.0043 (7)
O4	0.0300 (9)	0.0300 (10)	0.0297 (9)	0.0039 (7)	0.0190 (7)	−0.0033 (7)
N1	0.0174 (8)	0.0171 (9)	0.0152 (8)	−0.0018 (7)	0.0052 (7)	−0.0004 (7)
N2	0.0190 (9)	0.0125 (10)	0.0194 (9)	0.0006 (8)	0.0072 (7)	−0.0015 (7)
N3	0.0272 (10)	0.0163 (10)	0.0225 (9)	−0.0019 (8)	0.0058 (8)	0.0020 (8)
N4	0.0188 (9)	0.0277 (11)	0.0174 (9)	−0.0020 (8)	0.0063 (7)	−0.0013 (8)
C1	0.0150 (9)	0.0188 (11)	0.0113 (9)	−0.0003 (8)	0.0024 (7)	−0.0001 (8)
C2	0.0181 (10)	0.0142 (10)	0.0173 (10)	−0.0011 (8)	0.0035 (8)	−0.0003 (8)
C3	0.0205 (10)	0.0188 (11)	0.0162 (10)	−0.0045 (9)	0.0026 (8)	0.0037 (8)
C4	0.0160 (10)	0.0234 (12)	0.0120 (9)	−0.0024 (9)	0.0075 (8)	0.0008 (8)
C5	0.0182 (10)	0.0173 (11)	0.0152 (10)	0.0023 (8)	0.0029 (8)	−0.0026 (8)
C6	0.0182 (10)	0.0147 (10)	0.0148 (9)	−0.0012 (8)	0.0048 (8)	0.0009 (8)
C7	0.0172 (10)	0.0199 (11)	0.0127 (9)	0.0008 (8)	0.0020 (8)	−0.0018 (8)
C8	0.0160 (10)	0.0216 (12)	0.0143 (10)	0.0004 (8)	0.0031 (8)	−0.0027 (8)
C9	0.0187 (10)	0.0261 (12)	0.0163 (10)	0.0004 (9)	0.0065 (8)	−0.0025 (9)
C10	0.0168 (10)	0.0256 (12)	0.0146 (10)	−0.0032 (9)	0.0094 (8)	0.0001 (9)
C11	0.0251 (11)	0.0236 (12)	0.0229 (11)	−0.0064 (9)	0.0076 (9)	0.0018 (9)
C12	0.0228 (11)	0.0219 (12)	0.0191 (11)	0.0001 (9)	0.0093 (9)	−0.0025 (9)

### Geometric parameters (Å, °)

**Table:**

S1—C11	1.714 (2)	C3—H3A	0.9300
S1—C8	1.731 (2)	C4—C5	1.396 (3)
O1—N3	1.245 (2)	C5—C6	1.362 (3)
O2—N3	1.226 (2)	C5—H5A	0.9300
O3—N4	1.228 (2)	C6—H6A	0.9300
O4—N4	1.226 (2)	C7—C8	1.453 (3)
N1—C7	1.298 (3)	C7—C12	1.499 (3)
N1—N2	1.370 (3)	C8—C9	1.380 (3)
N2—C1	1.357 (3)	C9—C10	1.428 (3)
N2—H1N2	0.79 (3)	C9—H9A	0.9300
N3—C2	1.443 (3)	C10—C11	1.363 (3)
N4—C4	1.462 (3)	C10—H10A	0.9300
C1—C2	1.415 (3)	C11—H11A	0.9300
C1—C6	1.415 (3)	C12—H12A	0.9600
C2—C3	1.394 (3)	C12—H12B	0.9600
C3—C4	1.368 (3)	C12—H12C	0.9600
			
C11—S1—C8	91.98 (11)	C4—C5—H5A	120.4
C7—N1—N2	116.47 (18)	C5—C6—C1	121.80 (19)
C1—N2—N1	118.89 (18)	C5—C6—H6A	119.1
C1—N2—H1N2	116.4 (18)	C1—C6—H6A	119.1
N1—N2—H1N2	124.2 (18)	N1—C7—C8	114.90 (19)
O2—N3—O1	121.94 (18)	N1—C7—C12	124.81 (19)
O2—N3—C2	119.03 (18)	C8—C7—C12	120.30 (18)
O1—N3—C2	119.03 (17)	C9—C8—C7	128.3 (2)
O4—N4—O3	123.94 (18)	C9—C8—S1	110.63 (16)
O4—N4—C4	117.31 (18)	C7—C8—S1	121.11 (15)
O3—N4—C4	118.75 (18)	C8—C9—C10	112.89 (19)
N2—C1—C2	123.19 (19)	C8—C9—H9A	123.6
N2—C1—C6	119.84 (18)	C10—C9—H9A	123.6
C2—C1—C6	116.98 (18)	C11—C10—C9	112.11 (19)
C3—C2—C1	121.38 (19)	C11—C10—H10A	123.9
C3—C2—N3	116.11 (18)	C9—C10—H10A	123.9
C1—C2—N3	122.50 (18)	C10—C11—S1	112.39 (18)
C4—C3—C2	118.73 (19)	C10—C11—H11A	123.8
C4—C3—H3A	120.6	S1—C11—H11A	123.8
C2—C3—H3A	120.6	C7—C12—H12A	109.5
C3—C4—C5	121.90 (19)	C7—C12—H12B	109.5
C3—C4—N4	119.07 (19)	H12A—C12—H12B	109.5
C5—C4—N4	119.03 (19)	C7—C12—H12C	109.5
C6—C5—C4	119.21 (19)	H12A—C12—H12C	109.5
C6—C5—H5A	120.4	H12B—C12—H12C	109.5
			
C7—N1—N2—C1	178.22 (17)	C3—C4—C5—C6	−0.4 (3)
N1—N2—C1—C2	175.14 (17)	N4—C4—C5—C6	−179.49 (17)
N1—N2—C1—C6	−4.9 (3)	C4—C5—C6—C1	0.0 (3)
N2—C1—C2—C3	−179.90 (18)	N2—C1—C6—C5	−179.89 (18)
C6—C1—C2—C3	0.2 (3)	C2—C1—C6—C5	0.1 (3)
N2—C1—C2—N3	−1.2 (3)	N2—N1—C7—C8	178.99 (16)
C6—C1—C2—N3	178.88 (17)	N2—N1—C7—C12	−1.1 (3)
O2—N3—C2—C3	−1.9 (3)	N1—C7—C8—C9	177.69 (19)
O1—N3—C2—C3	178.29 (17)	C12—C7—C8—C9	−2.2 (3)
O2—N3—C2—C1	179.28 (19)	N1—C7—C8—S1	−2.2 (2)
O1—N3—C2—C1	−0.5 (3)	C12—C7—C8—S1	177.89 (15)
C1—C2—C3—C4	−0.5 (3)	C11—S1—C8—C9	−0.67 (16)
N3—C2—C3—C4	−179.26 (17)	C11—S1—C8—C7	179.21 (17)
C2—C3—C4—C5	0.6 (3)	C7—C8—C9—C10	−178.97 (19)
C2—C3—C4—N4	179.69 (17)	S1—C8—C9—C10	0.9 (2)
O4—N4—C4—C3	175.19 (18)	C8—C9—C10—C11	−0.7 (3)
O3—N4—C4—C3	−5.1 (3)	C9—C10—C11—S1	0.2 (2)
O4—N4—C4—C5	−5.7 (3)	C8—S1—C11—C10	0.27 (17)
O3—N4—C4—C5	174.08 (18)		

### Hydrogen-bond geometry (Å, °)

**Table:**

*D*—H···*A*	*D*—H	H···*A*	*D*···*A*	*D*—H···*A*
N2—H1N2···O1	0.79 (3)	2.00 (3)	2.618 (3)	134 (2)
C6—H6A···O2^i^	0.93	2.45	3.099 (3)	127
C9—H9A···O4^ii^	0.93	2.47	3.147 (2)	129
C11—H11A···O3^i^	0.93	2.57	3.240 (3)	129

Symmetry codes:  (i) −*x*+1, *y*−1/2, −*z*+3/2; (ii) *x*−1, *y*, *z*+1.
